# Poultry Farm Workers' Knowledge, Attitudes, and Practices on Antimicrobial Use and Resistance in Eastern Ethiopia: An Institutional‐Based Cross‐Sectional Study

**DOI:** 10.1002/hsr2.73054

**Published:** 2026-08-17

**Authors:** Mohamed Abdi, Jemal Mohammed, Ukash Umer, Gada Diba, Fitsum Weldegebreal

**Affiliations:** ^1^ Somali Regional State Pastoral Development Bureau Jigjiga Ethiopia; ^2^ Department of Microbiology and Veterinary Public Health College of Veterinary Medicine, University of Kabridahar Kabridahar Ethiopia; ^3^ School of Medical Laboratory Sciences, College of Health and Medical Sciences Haramaya University Harar Ethiopia; ^4^ Laboratory Bacteriology Research, Department of Diagnostic Sciences, Faculty of Medicine and Health Sciences Ghent University Ghent Belgium

**Keywords:** antimicrobial use and resistance, attitudes and practices, Ethiopia, knowledge, poultry farm workers

## Abstract

**Background and Aims:**

Poultry farming is a significant source of food worldwide; however, irrational antimicrobial use has contributed to antimicrobial resistance (AMR), posing a serious threat to humans, animals, and the environment. Despite this growing concern, the level of knowledge, attitudes, and practices (KAP) of poultry farm workers regarding antimicrobial use and resistance remains poorly understood in eastern Ethiopia. Therefore, this study aimed to assess poultry farm workers' KAP toward antimicrobial use and resistance, and to identify associated factors.

**Methods:**

An institution‐based cross‐sectional study was conducted among 414 randomly selected poultry farm workers from March 15 to April 27, 2025. Data were collected using a structured questionnaire through face‐to‐face interviews on ODK. Data were analyzed using STATA 14. Logistic regression analysis was carried out to examine the associations between dependent and independent variables. A *p*‐value of less than 0.05 was considered statistically significant.

**Results:**

About 53.62%, 48.31%, and 52.17% of participants had good knowledge, positive attitudes, and good practices on antimicrobial use and resistance, respectively. Educational status was significantly associated with knowledge (AOR = 2.3), attitudes (AOR = 3.5), and practices (AOR = 3.1). Similarly, farming experience was associated with knowledge (AOR = 2.6), attitudes (AOR = 4.4), and practices (AOR = 2.3). Source of information (AOR = 12.6) was associated with knowledge only, whereas access to veterinary services (AOR = 3.9) was associated with practices only.

**Conclusion:**

This study revealed significant gaps in participants' KAP regarding antimicrobial use and resistance. Education level and farming experience were significantly associated with KAP. Information source influenced knowledge, whereas availability of veterinary services influenced practices. Therefore, it is crucial to enhance poultry farm workers' KAP through implementing regular training programs that focus on the responsible use of antimicrobials and also through improving access to poultry health information and services to minimize AMR challenges.

**Abbreviations**
AMR: antimicrobial resistance
AMU: antimicrobial use
AOR: adjusted odd ratio
CI: confidence interval
COR: crude odd ratio
IHRERC: Institutional Health Research and Ethics Review Committee
KAP: knowledge, attitudes and practices
LMICs: low and middle income countries
ODK: Open Data Kit
VIF: variance inflation factor

## 1 Introduction

Poultry farming is a globally important industry, providing food for billions of people worldwide, with developing countries leading in production of millions of metric tons of poultry products each year [1]. Antimicrobials are used in animal farming, including poultry, to prevent and treat diverse infections. However, the expansion of poultry farming methods has resulted in the extensive utilization of antimicrobials for disease prevention, treatment, prophylaxis, and growth promotion, leading to the rise of antimicrobial resistance (AMR) [2]. This phenomenon occurs when microorganisms develop the ability to resist the effects of drugs designed to kill them, which poses a significant threat to public health [3]. The overuse and misuse of antimicrobials in food animal production, including poultry, are suggested to be the major driver of AMR, as it promotes the emergence and spread of resistant pathogens that can affect human health through the food chain or direct contact with animals [4, 5].

AMR is a 1 of 10 global health crises, affecting human, animal, and environmental health [6]. Poultry farming, particularly in low‐ and middle‐income countries (LMICs), plays a crucial role in the emergence and spread of AMR due to the widespread and often inappropriate use of antimicrobials [7]. The increasing focus on intensive poultry farming practices, often with limited biosecurity measures, can potentially facilitate the emergence and spread of resistant pathogens [8]. Weak regulatory frameworks and inadequate regulations on antimicrobial use (AMU) in food animals can also contribute to the problem [9].

In Ethiopia, the prevalence of AMR among poultry pathogens is a pressing concern. Notably, a study done in the southern part of the country found that 100% of *Salmonella* isolates from poultry breeding centers exhibited resistance to commonly used antimicrobials [10]. Similarly, a considerable proportion (96.8%) of *Salmonella* isolates from poultry products in central Ethiopia displayed resistance to various antimicrobials, emphasizing the critical need to tackle AMR in the country's poultry production [11]. Additionally, previous studies carried out in north and northwest Ethiopia revealed the overall multidrug‐resistant rates of 79.1% and 81.5%, respectively, among poultry production [12, 13].

Several factors are contributing to the emergence and expansion of AMR in animal farming, including poultry. These are: limited knowledge related to AMU and AMR, inadequate biosecurity practices, limited access to veterinary services, and the availability and affordability of antimicrobials [14]. Additionally, failure to observe withdrawal periods before slaughtering animals can result in the transmission of antimicrobial‐resistant pathogens to humans [14].

Having information about farm owners/workers' knowledge, attitudes, and practices (KAP) regarding AMU and resistance is crucial since it could aid in the development of strategies that maximize and preserve the benefits of AMU in animal production while posing the least risk to public health. Moreover, it may also enable policy‐makers and regulatory bodies to design targeted intervention and management strategies [15]. However, there is no prior study on the KAP of animal farm workers regarding AMU and resistance in eastern Ethiopia. Thus, this study aimed to assess poultry farm workers' KAP on AMU, resistance, and associated factors in eastern Ethiopia.

## 2 Materials and Methods

### 2.1 Study Setting

The study was conducted in three cities of eastern Ethiopia (Jigjiga, Harar, and Dire Dawa) from March 15 to April 27, 2025. These cities were selected due to their existing highly poultry‐farmed farms, proximity to essential water sources for poultry, and good access to markets. These factors make them attractive locations for poultry farming, which is a significant source of income for many residents.

Harar, the capital city of the Harari Regional State, is located 526 km east of Addis Ababa [16]. According to the Harar City Agricultural Development Office (2025), the city had 50 poultry farms with 162 workers and 54,835 chickens. Dire Dawa, situated 515 km east of Addis Ababa [17], had 44 poultry farms with 149 workers and 68,715 chickens (Dire Dawa Agricultural and Livestock Development Office, 2025). Jigjiga, the capital city of the Somali Regional State, is located 630 km southeast of Addis Ababa [16], and had 145 poultry farms with 440 workers and 120,939 chickens (Jigjiga Agricultural, Livestock, and Environmental Protection Office, 2025).

### 2.2 Study Design and Population

An institution‐based cross‐sectional study was conducted. All poultry farm workers who were working at all poultry farms found in those three selected cities were the source population. The study population comprised all poultry farm workers who were working at poultry farms with flocks of small‐scale and above during the study period. Poultry farm workers who had at least 1 year of farming experience and volunteered to participate in the study were included in the study. In contrast, poultry farm workers working at poultry farms with extensive scavenging systems, involving flocks of fewer than 100 indigenous or crossbred chickens, and typically access to rural markets were excluded from the study.

### 2.3 Sample Size Determination and Sampling Procedure

The sample size was calculated using a single population proportion formula, assuming a 50% level of KAP among poultry farm workers regarding AMU and resistance, due to the absence of prior studies in the area. A 95% confidence interval, 5% margin of error, and 10% non‐response rate were considered, yielding a final sample size of 422 participants. In terms of the sampling procedure, there were 239 eligible poultry farms with 751 farm workers and 244,489 chickens across the three cities. To enroll poultry farm workers from each city in the study, a simple random sampling technique was used by considering a list (name) of poultry farm workers on separate Excel sheets as a sampling frame. Then, the calculated sample size was proportionally allocated to each city based on their respective number of poultry farm workers.

### 2.4 Data Collection Methods

First, a structured questionnaire was developed in English after reviewing relevant different literature [18, 19, 20, 21]. Then, it was translated to local languages (Afan Oromo, Amharic, and Af‐Somali) and again re‐translated back to English to maintain its consistency by language experts who were fluent in those three languages. Data on socio‐demographic, behavioral, farm hygiene management, and KAP level‐related factors were collected by six trained BSc veterinarians (two for each city) using a pre‐tested structured questionnaire through face‐to‐face interviews under the supervision of three senior BSc public health professionals and microbiologists with extensive experience in food safety and AMR.

### 2.5 Operational Definitions

We defined KAP by considering cut‐off points from similar previous studies conducted elsewhere to dichotomize the KAP scores as shown below.

#### 2.5.1 Knowledge

Knowledge was defined as a participant's score on eight questions, with each question having 1 point. A score of 4 or more correct answers indicates “good knowledge”, while a score of three or fewer correct answers indicates “poor knowledge” [21].

#### 2.5.2 Attitude

Attitude was defined as a participant's cumulative score on 14 Likert‐scale questions (14 × 5 = 70). Each question is scored from 1 (*strongly disagree*) to 5 (*strongly agree*) points for each category. A total score ranging from 35 to 70 points indicates a “positive attitude”, while a total score ranging from 14 to 34 points indicates a “negative attitude” [22].

#### 2.5.3 Practice

Practice was defined as a participant's score on nine questions (self‐reported practice test), with each question having one point. A total score ranging from 5 to 9 points indicates “good practice”, while a total score ranging from 0 to 4 points indicates “poor practice” [23].

### 2.6 Data Processing and Analysis

Data were collected using the Open Data Kit (ODK) digital platform, and then, it was exported to STATA version 14.0 for cleaning and analysis. Descriptive statistical analysis was performed to descriptively summarize the data. Bi‐variable and multivariable logistic regression analysis was used to assess the association of independent variables with KAP of participants on AMU and AMR. Independent variables with *p*‐value less than or equal to 0.25 with each of the three KAP domains in bi‐variable analysis were considered as candidate variables for multivariable logistic regression analysis to control the possible effect of confounders. A variance inflation factor (VIF) was used to examine the degree of multicollinearity. Moreover, the model goodness of fit was checked using the Hosmer‐Lemeshow statistic; the model was considered a good fit if it was found to be insignificant for the Hosmer‐Lemeshow statistic (> 0.05). The association between KAP scores and independent variables was reported as an adjusted odds ratio (AOR) with its 95% CI, and a two‐sided *p*‐value < 0.05 in the final model was considered statistically significant.

### 2.7 Data Quality Assurance

Two days of training were given for data collectors and supervisors on the aims of the study, detailed contents and procedures of the data collection tool by the principal investigator. The principal investigator also regularly monitored the data collection, ensuring that the collected data were complete and consistent. Moreover, the questionnaire was pre‐tested on 5% of the total sample at Shabelay town, Somali region, who were not included in this study, 1 week prior to the actual data collection. Then, based on the pre‐test results, the required modifications and changes were made.

### 2.8 Ethical Consideration

Ethical clearance was obtained from Haramaya University, College of Health and Medical Sciences, Institutional Health Research Ethics Review Committee (IHRERC) (reference number: IHRERC/030/2025). A cooperation letter was obtained from the respective agricultural and livestock offices of the selected cities and subsequently submitted to each selected poultry farm. Before the interview, to obtain informed, voluntary written consent, all study participants were informed about the study's objectives, risks, benefits, and confidentiality issues, as well as their right to participate or not. The respondents were assured that the data obtained from them were kept strictly confidential (i.e., unique codes were used rather than any personal identifiers or names, and only authorized personnel had access to the collected data) and were not used for any purpose other than this study.

## 3 Results

### 3.1 Sociodemographic and Behavioral Characteristics

A total of 414 poultry farm workers participated in this study with a response rate of 98.1%. A total of 242 (58.5%), 83 (20.0%), and 89 (21.5%) of participants were from Jigjiga, Dire Dawa, and Harar city, respectively. A total of 306 (73.9%) and 216 (52.2%) of participants were working in layer production and small scale farm, respectively. A total of 314 (75.9%) of participants were males. Almost equal numbers, 169 (40.8%) and 166 (40.1%), of respondents belonged to the age group of 18–30 and 31–45 years, respectively. Regarding educational status, 140 (33.8%) of respondents reported that they attended college and above. About 254 (61.4%) of participants had less than or equal to 5 years of poultry farming experience. Moreover, 305 (73.7%) of respondents reported that they routinely use antimicrobials, even when their poultry appeared healthy; of these, 48.2% of them mainly used them for treatment purposes (Table 1).

**Table 1:** Sociodemographic and behavioral characteristics of participants in eastern Ethiopia, 2025 (*n* = 414).

Variables	Categories	Frequency (*n*)	Percent (%)
Farm location	Jigjiga	242	58.5
	Dire Dawa	83	20.0
	Harar	89	21.5
Production type	Layer	306	73.9
	Broiler	32	7.7
	Dual	76	18.4
Farm size	Small scale	216	52.2
	Medium scale	168	40.6
	Large scale	30	7.3
Sex	Male	314	75.9
	Female	100	24.2
Age (in years)	18–30	169	40.8
	31–45	166	40.1
	≥ 46	79	19.1
Educational status	Unable to read and write	27	6.5
	Able to read and write	75	18.1
	Primary school	87	21.0
	Secondary school	85	20.5
	College and above	140	33.8
Farm experience (in years)	≤ 5	254	61.4
	6–15	134	32.4
	≥ 16	26	6.3
Antimicrobial use, even when the poultry appear healthy	Yes	305	73.7
	No	109	26.3
Reason for antimicrobial usage	Treatment	147	48.2
	Increase production	53	17.4
	Prevention (prophylaxis)	59	19.3
	Control (metaphylaxis)	46	15.1
Access to veterinary services	Private pharmacy without prescription	229	55.3
	Health posts or clinics	66	15.9
	Market centers	119	28.7
Source of information about poultry health	Veterinarians	150	36.2
	Extension officer	163	39.4
	Poultry farmers	94	22.7
	Social media	7	1.7

### 3.2 Knowledge on AMU and Resistance

In this study, most of the study participants (91.6%) were familiar with antibiotics or antimicrobials. A total of 249 (60.1%) of participants reported that they had information about AMU and AMR; of which, the majority (70.7%) of them had heard about it from veterinarians. A total of 144 (34.8%), 130 (31.4%), and 158 (38.2%) of participants did not know that incomplete dose, over‐dose, and under‐dose use of antimicrobials may lead to AMR, respectively. Similarly, 206 (49.8%) of respondents reported that they did not know about antimicrobial residues. About 58.5% of study subjects had information about the antibiotic withdrawal period. Overall, 53.6% (95% CI: 48.7, 58.5) of study subjects had good knowledge of AMU and AMR (Table 2).

**Table 2:** Knowledge of the poultry farm workers on antimicrobial use and resistance in eastern Ethiopia, 2025 (*n* = 414).

Variables	Categories	Frequency (*n*)	Percent (%)
Know about antibiotics or antimicrobials	Yes	379	91.6
	No	35	8.5
Heard about AMU and AMR	Yes	249	60.1
	No	165	39.9
Sources of health information	Veterinarians	176	70.7
	Agricultural extension	52	20.9
	Poultry farmers	19	7.6
	Social Media	2	0.8
Know about incomplete antibiotic use may lead to AMR	Yes	150	36.2
	No	120	29.0
	I don't know	144	34.8
Know overdose of antimicrobial may lead to AMR	Yes	184	44.5
	No	100	24.2
	I don't know	130	31.4
Know under‐dose of antimicrobial may lead to AMR	Yes	102	24.6
	No	154	37.2
	I don't know	158	38.2
Know about antimicrobial residues	Yes	208	50.2
	No	206	49.8
Heard about withdrawal periods of antimicrobials	Yes	242	58.5
	No	172	41.6
Knowing of not observing withdrawal period leads to AMR	Yes	180	74.4
	No	7	2.9
	I don't know	55	22.7
Know using animal‐origin food products before the end of the withdrawal period can promote AMR development in humans?	Yes	114	27.5
	No	62	15.0
	I don't know	238	57.5
Overall knowledge level	Good	222	53.6
	Poor	192	46.4

### 3.3 Attitudes Towards AMU and Resistance

The study revealed considerable gaps and ambivalence in participants' attitudes towards AMU and AMR in poultry. Only 33.1% of participants recognized antibiotic resistance in poultry as a significant public health concern, while less than one‐third (29.71%) acknowledged the relationship between antibiotic use in poultry and the development of AMR. Regarding disease prevention, 28.7% of participants expressed a neutral stance on the use of antimicrobials for protection against diseases. Notably, 28.3% of participants agreed that restricting antibiotic use in animals would yield more benefits than harm. Awareness of the risks posed by improper AMU was limited, with only 25.4% of respondents agreeing that antimicrobial residues and resistance could result from imprudent use (Table 3).

**Table 3:** Attitudes of poultry farm workers towards antimicrobial use and resistance in eastern Ethiopia, 2025 (*n* = 414).

Variables	Categories: *n* (%)				
	Strongly agree	Agree	Neutral	Disagree	Strongly disagree
AMR in poultry is a concern for public health	129 (31.2)	137 (33.1)	77 (18.6)	49 (11.8)	22 (5.3)
There is a link between AMU in poultry and the development of AMR	66 (15.9)	123 (29.7)	94 (22.7)	99 (23.9)	32 (7.7)
AMU for protection against diseases is crucial	63 (15.2)	110 (26.6)	119 (28.7)	90 (21.7)	32 (7.7)
Restriction of AMU in animals will lead to more benefit than damage	44 (10.6)	117 (28.3)	100 (24.2)	106 (25.6)	47 (11.4)
AMR and residues will occur when antimicrobials are not used prudently	39 (9.4)	105 (25.4)	127 (30.7)	103 (24.9)	40 (9.7)
Misuse of antibiotics in poultry causes the emergence of resistant bacteria, which cause diseases in humans	59 (14.3)	112 (27.1)	141 (34.1)	88 (21.3)	14 (3.4)
When using antibiotics properly, there is less risk of AMR.	39 (9.4)	99 (23.9)	140 (33.8)	91 (22.0)	45 (10.9)
Stopping AMU once poultry feel better leads to AMR	37 (8.9)	84 (20.3)	140 (33.8)	101 (24.4)	52 (12.6)
Drug withdrawal periods should be adhered to as per the prescription to avoid drug residues	37 (8.9)	110 (26.6)	134 (32.4)	97 (23.4)	36 (8.7)
Sale and distribution of Antimicrobials shall only be done by authorized personnel	36 (8.7)	101 (24.4)	135 (32.6)	95 (23.0)	47 (11.4)
AMU may be reduced by proper biosecurity, vaccination, and good management	45 (10.9)	94 (22.7)	136 (32.9)	92 (22.2)	47 (11.4)
Antibiotics are used only when needed	42 (10.1)	92 (22.2)	126 (30.4)	103 (24.9)	51 (12.3)
Antibiotics should be prescribed only by veterinarians	41 (9.9)	93 (22.5)	113 (27.3)	120 (29.0)	47 (11.4)
AMU for non‐therapeutic reasons leads to AMR	43 (10.4)	96 (23.2)	102 (24.6)	88 (21.3)	85 (20.5)
Overall attitude level	Positive				200 (48.3)
	Negative				214 (51.7)

Concerning the link between antibiotic misuse in poultry and the emergence of resistant bacteria that cause human disease, merely 27.1% of participants agreed with this connection. Similarly, 23.9% of study subjects acknowledged that proper antibiotic use reduces the risk of resistance. When asked about the practice of discontinuing antimicrobial treatment once poultry appeared healthy, 33.8% of participants remained neutral. Furthermore, 32.4% were neutral about adherence to drug withdrawal periods to prevent residues in poultry products. On regulatory issues, only 24.4% of participants agreed that the sale and distribution of antimicrobials should be restricted to authorized personnel. Participants also showed uncertainty regarding alternative AMU reduction strategies; 32.9% of respondents were neutral about reducing antibiotic use through improved bio‐security, vaccination, and management. Notably, 29.0% of participants disagreed that antibiotics should be prescribed exclusively by veterinarians. Additionally, only 33.6% of workers agreed that the non‐therapeutic use of antibiotics contributes to AMR development. Overall, 48.3% (95% CI: 43.4, 53.2) of farm workers had a positive attitude towards AMU and AMR (Table 3).

### 3.4 Practices Regarding AMU and Resistance

The findings indicate that a substantial proportion of participants (75.8%) reported administering antibiotics to sick poultry without prior consultation with a veterinarian. Furthermore, 71.5% of respondents expressed unwillingness to conduct laboratory testing before selecting and using antimicrobials. While a majority (87.9%) of participants reported checking the expiration dates of antibiotics, only 58.9% of workers read product descriptions prior to use. Adherence to recommended withdrawal periods was notably low; only 33.1% of participants followed the specified withdrawal period before selling poultry for slaughter. Additionally, 64.7% of study subjects reported applying manure from antibiotic‐treated poultry as a natural fertilizer before the withdrawal period had concluded, potentially contributing to environmental contamination and AMR. Overall, 52.2% (95% CI: 47.2, 57.1) of participants demonstrated good practices on AMU and AMR (Table 4).

**Table 4:** Practices of the poultry farm workers on antimicrobial use and resistance in eastern Ethiopia, 2025 (*n* = 414).

Variables	Categories	Frequency (*n*)	Percent (%)
Prescribed antibiotics to sick poultry without prior consultation with a veterinarian	Yes	314	75.8
	No	100	24.2
Consult a veterinarian whether they need to use antibiotics or not	Yes	240	58.0
	No	174	42.0
Readiness for laboratory test before choosing and using antimicrobial	Yes	118	28.5
	No	296	71.5
Check expiry date of antibiotics before using	Yes	364	87.9
	No	50	12.1
Read product description before using antimicrobial	Yes	244	58.9
	No	170	41.1
Follow specified drug withdrawal period before selling the poultry for slaughter	Yes	137	33.1
	No	277	66.9
Increase dose and frequency of antibiotics as long as poultry do not show any signs of recovery	Yes	262	63.3
	No	152	36.7
Stop giving the antibiotics if poultry feel better before the antibiotic course is completed	Yes	300	72.5
	No	114	27.5
Use treated‐poultry manure as a fertilizer before the recommended withdrawal period is over	Yes	268	64.7
	No	146	35.3
Overall practice level	Good	216	52.2
	Poor	198	47.8

### 3.5 Factors Associated With Knowledge of Poultry Farm Workers

In bivariate analysis, variables such as farm location, farm size, sex, educational status, farming experience, and source of information were found to be significantly associated with knowledge. However, during the multivariable logistic regression analysis, only educational status, farming experience, and source of information remained significantly associated with knowledge (Table 5).

**Table 5:** Factors associated with knowledge of poultry farm workers on antimicrobial use and resistance in eastern Ethiopia, 2025.

Variables	Categories	Overall knowledge		COR (95% CI)	*p*‐value	AOR (95% CI)	*p*‐value
		Good (%)	Poor (%)				
Farm location	Jigjiga	114 (47.1)	128 (52.9)	1		1	
	Dire Dawa	62 (74.7)	21 (25.3)	3.3 (1.90–5.80)	0.001	0.6 (0.27–1.53)	0.313
	Harar	46 (51.7)	43 (48.3)	1.2 (0.73–1.96)	0.460	0.2 (0.07–1.42)	0.321
Production type	Layer	162 (52.9)	144 (47.1)	1			
	Broiler	19 (59.4)	13 (40.6)	1.3 (0.62–2.72)	0.488	—	
	Dual	41 (53.9)	35 (46.1)	1.1 (0.63–1.72)	0.875		
Farm size	Small scale	102 (47.2)	114 (52.8)	1		1	
	Medium scale	98 (58.3)	70 (41.7)	1.6 (1.04–2.35)	0.031	0.9 (0.54–1.83)	0.991
	Large scale	22 (73.3)	8 (26.7)	3.1 (1.31–7.20)	0.010	0.4 (0.11–1.45)	0.165
Sex	Female	31 (31.0)	69 (69.0)	1		1	
	Male	191 (60.8)	123 (39.2)	3.4 (2.14–5.59)	0.001	1.9 (0.97–3.65)	0.060
Age (in years)	18–30	87 (51.5)	82 (48.5)	1			
	31–45	94 (56.6)	72 (43.4)	1.2 (0.80–1.90)	0.345	—	
	≥ 46	41 (51.9)	38 (48.1)	1.1 (0.59–1.73)	0.951		
Educational status	Unable to read and write	1 (3.7)	26 (96.3)	1		1	
	Able to read and write	17 (22.7)	58 (77.3)	7.6 (2.96–13.34)	0.054	4.1 (0.23–10.52)	0.520
	Primary	29 (33.3)	58 (66.7)	1.7 (1.68–19.62)	0.014	2.2 (0.70–14.84)	0.100
	Secondary	56 (65.9)	29 (34.1)	3.9 (1.42–18.54)	0.001	4.0 (1.03–16.74)	0.005
	College and above	119 (85.0)	21 (15.0)	2.9 (1.96–9.45)	0.000	2.3 (1.81–8.32)	0.001
Farming experience	≤ 5 yrs	112 (44.1)	142 (55.9)	1		1	
	6–15 yrs	91 (67.9)	43 (32.1)	2.6 (1.73–7.16)	0.001	2.6 (1.39–5.86)	0.003
	≥ 16 yrs	19 (73.1)	7 (26.9)	3.4 (1.40–8.48)	0.007	1.7 (0.47–6.27)	0.419
Source of information	Poultry farmers	13 (13.9)	81 (86.1)	1		1	
	Extension	77 (47.2)	86 (52.8)	5.6 (2.88–10.81)	0.001	3.2 (1.51–7.03)	0.003
	Veterinarians	128 (85.3)	22 (14.7)	36.2 (17.30–75.98)	0.000	12.6 (5.19–30.58)	0.001
	Social media	4 (57.1)	3 (42.9)	8.3 (1.6–41.45)	0.010	2.9 (0.38–23.21)	0.297

Participants with a secondary education were four times (AOR = 4.0, 95% CI: 1.03–16.74) more likely, and those with college and above education were 2.3 times (AOR = 2.3, 95% CI: 1.81–8.32) more likely to have good knowledge compared to those who were unable to read and write. The odds of having good knowledge were 2.6 times (AOR = 2.6, 95% CI: 1.39–5.86) higher among participants who had 6–15 years of farming experience compared with those who had 5 or less years of farming experience. Moreover, the odds of having good knowledge were 3.2 times (AOR = 3.2, 95% CI: 1.51–7.03) and 12.6 times (AOR = 12.6, 95% CI: 5.19–30.58) higher among participants who received health information from extension officers/professionals and veterinarians, respectively, compared with those who obtained information from poultry farmers (Table 5).

### 3.6 Factors Associated With Attitudes of Poultry Farm Workers

According to the bivariate analysis, variables such as farm location, farm size, sex, educational status and farming experience were found to be significantly associated with attitudes among poultry farm workers. However, in the multivariable analysis, only educational status and farming experience remained significantly associated with attitudes (Table 6).

**Table 6:** Factors associated with attitudes of poultry farm workers towards antimicrobial use and resistance in eastern Ethiopia, 2025.

Variables	Categories	Overall attitude level		COR (95% CI)	*p*‐value	AOR (95% CI)	*p*‐value
		Positive (%)	Negative (%)				
Farm location	Jigjiga	98 (40.5)	144 (59.5)	1		1	
	Dire Dawa	57 (68.7)	26 (31.3)	3.2 (1.89–5.47)	0.001	0.5 (0.23–1.24)	0.147
	Harar	45 (50.6)	44 (49.4)	1.5 (0.92–2.45)	0.102	0.3 (0.09–0.50)	0.158
Production type	Layer	151 (49.3)	155 (50.7)	1			
	Broiler	15 (46.9)	17 (53.1)	0.9 (0.43–1.88)	0.790	—	
	Dual	34 (44.7)	42 (55.3)	0.8 (0.5–1.38)	0.472		
Farm size	Small scale	94 (43.5)	122 (56.5)	1		1	
	Medium scale	83 (49.4)	85 (50.6)	1.2 (0.84–1.90)	0.251	0.8 (0.49–1.60)	0.679
	Large scale	23 (76.7)	7 (23.3)	4.3 (1.75–10.36)	0.001	1.5 (0.45–4.93)	0.504
Sex	Female	32 (32.0)	68 (68)	1		1	
	Male	168 (53.5)	146 (46.5)	2.4 (1.52–3.93)	0.001	1.1 (0.50–1.98)	0.958
Age (in years)	18–30	81 (48.0)	88 (52.0)	1			
	31–45	81 (48.8)	85 (51.2)	1.1 (0.67–1.59)	0.874	—	
	≥ 46	38 (48.1)	41 (51.9)	1.1 (0.59–1.72)	0.980		
Educational status	Unable to read and write	1 (3.7)	26 (96.3)	1		1	
	Able to read and write	10 (13.3)	65 (86.7)	4.0 (0.49–7.84)	0.197	2.9 (0.34–7.07)	0.330
	Primary	19 (21.9)	68 (78.1)	1.8 (0.92–10.05)	0.059	1.2 (1.18–11.85)	0.035
	Secondary	49 (57.6)	36 (42.4)	4.9 (1.59–13.01)	0.001	5.2 (1.00–9.23)	0.002
	College and above	121 (86.4)	19 (13.6)	4.7 (1.50–10.7)	0.000	3.5 (1.92–8.63)	0.001
Farm experience (in years)	≤ 5	97 (38.2)	157 (61.8)	1		1	
	6–15	85 (63.4)	49 (36.6)	2.8 (1.82–4.33)	0.001	2.8 (1.58–5.25)	0.001
	≥ 16	18 (69.2)	8 (30.8)	3.6 (1.52–8.70)	0.004	4.4 (1.20–16.26)	0.026

Participants with primary education were 1.2 times (AOR = 1.2, 95% CI: 1.18–11.85), those with secondary education were 5.2 times (AOR = 5.2, 95% CI: 1.00–9.23), and those with college and above education were 3.5 times (AOR = 3.5, 95% CI: 1.92–8.63) more likely to have a positive attitude compared to those who were unable to read and write. Regarding farming experience, participants who had 6–15 years of experience were 2.8 times (AOR = 2.8, 95% CI: 1.58–5.25), and those with 16 or more years were 4.4 times (AOR = 4.4, 95% CI: 1.20–16.26) more likely to have a positive attitude compared to those with 5 or less years of experience (Table 6).

### 3.7 Factors Associated With Practices of Poultry Farm Workers

In the bi‐variable analysis, variables such as farm location, farm size, sex, educational status, farming experience and availability of veterinary services were found to be significantly associated with practice. But, in the multivariable analysis, only educational status, farming experience, and availability of veterinary services remained significantly associated with practice (Table 7).

**Table 7:** Factors associated with practices of poultry farm workers on antimicrobial use and resistance in eastern Ethiopia, 2025.

Variables	Categories	Overall practice level		COR (95% CI)	*p*‐value	AOR (95% CI)	*p*‐value
		Good (%)	Poor (%)				
Farm location	Jigjiga	102 (42.1)	140 (57.9)	1		1	
	Dire Dawa	57 (68.7)	26 (31.3)	3 (1.78–5.10)	0.001	0.9 (0.51–1.98)	0.991
	Harar	57 (64)	32 (36)	2.4 (1.48–4.04)	0.001	0.9 (0.49–1.96)	0.958
Production type	Layer	162 (52.9)	144 (47.1)	1			
	Broiler	17 (53.1)	15 (46.9)	1.1 (0.48–2.08)	0.984	—	
	Dual	37 (48.7)	39 (51.3)	0.8 (0.51–1.40)	0.506		
Farm size	Small scale	105 (48.6)	111 (51.4)	1		1	
	Medium scale	89 (53.0)	79 (47.0)	1.2 (0.79–1.78)	0.396	1.1 (0.70–1.95)	0.556
	Large scale	22 (73.3)	8 (26.7)	2.9 (1.24–6.81)	0.014	1.6 (0.61–4.59)	0.312
Sex	Female	44 (44.0)	56 (56.0)	1		1	
	Male	172 (54.8)	142 (45.2)	1.5 (0.98–2.43)	0.061	0.7 (0.43–1.42)	0.423
Age (in years)	18–30	85 (50.3)	84 (49.7)	1			
	31–45	95 (57.2)	71 (42.8)	1.3 (0.86–2.03)	0.290	—	
	≥ 46	36 (45.6)	43 (54.4)	0.8 (0.48–1.42)	0.488		
Educational status	Unable to read and write	1 (3.7)	26 (96.3)	1		1	
	Able to read and write	19 (25.3)	56 (74.7)	8.8 (5.12–13.49)	0.039	4.2 (0.98–18.44)	0.052
	Primary	38 (43.7)	49 (56.3)	2.3 (1.62–11.34)	0.004	2.4 (1.68–14.71)	0.004
	Secondary	48 (56.4)	37 (43.6)	1.7 (1.38–12.13)	0.001	2.9 (1.30–13.24)	0.002
	College and above	110 (78.5)	30 (21.5)	2.8 (1.42–10.5)	0.001	3.1 (1.31–12.80)	0.001
Farm experience	≤ 5 years	111 (43.7)	143 (56.3)	1		1	
	6–15 years	91 (67.9)	43 (32.1)	2.7 (1.76–4.23)	0.001	2.3 (1.42–4.05)	0.001
	≥ 16 years	14 (53.8)	12 (46.2)	1.5 (0.67–3.38)	0.324	0.8 (0.28–2.35)	0.702
Access to veterinary services	Private pharmacy without prescription	102 (44.5)	127 (55.5)	1		1	
	Animal health posts or clinics	52 (78.8)	14 (21.2)	4.6 (2.42–8.81)	0.001	3.9 (1.87–8.37)	0.001
	Market centers	62 (52.1)	57 (47.9)	1.35 (0.87–2.12)	0.181	1.1 (0.69–2.04)	0.524

Participants with primary education were 2.4 times (AOR = 2.4, 95% CI: 1.68–14.71), those with secondary education were 2.9 times (AOR = 2.9, 95% CI: 1.30–13.24), and those with college and above education were 3.1 times (AOR = 3.1, 95% CI: 1.31–12.80) more likely to demonstrate good practices on AMU and AMR compared to those who were unable to read and write. The odds of demonstrating good practices were 2.3 times (AOR = 2.3, 95% CI: 1.42–4.05) higher among participants who had 6–15 years of farming experience compared with those who had 5 years or less experience. Regarding veterinary services, participants who accessed services through animal health posts or clinics were 3.9 times (AOR = 3.9, 95% CI: 1.87–8.37) more likely to demonstrate good practices compared to those who accessed services or medication from private pharmacies without prescription (Table 7).

## 4 Discussion

The study was conducted to assess the KAPs of poultry farm workers concerning AMU, AMR, and their associated factors in selected cities of eastern Ethiopia. Findings revealed that 53.6% (95% CI: 48.7, 58.5) of the participants had good knowledge, 48.3% (95% CI: 43.4, 53.2) exhibited a positive attitude, and 52.2% (95% CI: 47.2, 57.1) reported good practices related to AMU and AMR. The analysis also showed that workers' KAPs were significantly linked to their education level and farming experience. In addition, access to information sources was associated only with their knowledge, whereas the availability of veterinary services was linked only to their practices.

In the present study, a notable knowledge gap was observed, with only 53.6% of poultry farm workers demonstrating good knowledge regarding AMU and AMR. This finding was consistent with the studies conducted in Kitwe, Zambia (53.8%) [21], Tanzania (55.5%) [24], Addis Ababa, Ethiopia (57.7%) [22], and the Amhara region, northwestern Ethiopia (50.0%) [15] and Cameroon (53.3%) [25]. However, the level of knowledge reported in this study was higher than that reported from Bangladesh (41.5%) [26], Vietnam (42.1%) [19], yet lower than findings reported from Kellem Wollega, Ethiopia (66.5%) [27] and Malaysia (60.8%) [28]. This variation could be due to differences in the existence of more intensive commercial farming operations, information accessibility, or local awareness campaigns [20]. In addition, the possible justification for the observed low‐level knowledge might be due to the study participants' lower levels of educational status, limited farm experience, and a reliance on unprofessional information sources concerning poultry health.

In the current study, educational status was found to be significantly associated with knowledge about AMU and AMR. This result was in agreement with a previous study done in Oromia, Ethiopia [29], Addis Ababa, and Vietnam [19]. This may be due to the fact that good knowledge is associated with education because farmers/workers with a higher level of education are better able to understand the consequences of improper use of antibiotics [14].

In this study, farming work experiences significantly associated with knowledge on AMU and AMR. This result was in line with the studies conducted in Rwanda [30], Addis Ababa [22], and East Wollega zone, Ethiopia [31] that found knowledge was positively influenced by workers' farm experience. This finding might indicate that practical experience is also more likely to provide knowledge about AMU and AMR.

In the current study, access to health information was found to be significantly associated with knowledge on AMU and AMR. This finding was consistent with previous studies done in Tanzania [24], East Wollega zone, Ethiopia [31], and Bangladesh [32]. This could be explained by the fact that getting information from veterinarians is linked to having good knowledge because they offer comprehensive guidance on the use of antimicrobials [33].

In the current study, the level of positive attitudes of poultry farm workers towards AMU and AMR was 48.3%. This finding was consistent with the studies conducted in Thailand (49.0%) [34]and northwestern Ethiopia (52.5%) [15]. However, the level of positive attitude reported in this study was higher than that reported from Bangladesh (42.5%) [26], Oromia region, Ethiopia (15.7%) [29], Kitwe, Zambia (28.3%) [21]; yet lower than the findings from Nepal (91.6%) [35]. These variations might be due to differences in educational level, farming experience, and the scope and effectiveness of public health initiatives [33].

In this study, educational status was found to be significantly associated with attitudes toward AMU and AMR. This result was in line with previous studies conducted in Uganda [36] and Bangladesh [26]. This suggests that education plays a key role in shaping attitudes, as higher education is associated with greater understanding of health‐related issues.

In the current study, farming work experiences were significantly associated with attitudes toward AMU and AMR. This finding was supported by previous studies done in Addis Ababa [22], East Wollega zone, Ethiopia [31], and Rwanda [30]. This could be explained by the fact that having more years of farming work experience is linked to more positive attitudes towards AMU and AMR.

In this study, the good practices level of poultry farm workers on AMU and AMR was 52.2%. This finding was comparable to previous studies done in Addis Ababa (53.0%) [22], Rwanda (52.8%) [30], Tanzania (50.9%) [24], and northwestern Ethiopia (47.2%) [15]. However, the level of good practices reported in this study was higher than that reported in Bangladesh (21.7%) [26] and Ethiopia (27.7%) [37]; yet lower than findings from Kellem Wollega (61.7%) [27]. The observed variations in findings across different regions could be due to differences in local awareness campaigns, information accessibility, and access to veterinary services, which may have an impact on practice levels [32]. The low practice coverage found in the present study could be due to participants' lower education levels, limited farm experience, and insufficient access to formal veterinary services like animal health post/clinics.

In the current study, poultry farm workers with primary and above level of education were more likely to have good practices compared to those who were unable to read and write. This result was consistent with previous studies conducted in Zambia [38], Kiambu, Kenya [39], KwaZulu‐Natal, South Africa [40], and Addis Ababa [22]. Higher education levels are linked to good practices because it helps farmers better understand and follow guidelines for drug use [38].

In the present study, farming work experiences were found to be significantly associated with practices regarding AMU and AMR. This result was in agreement with studies done in Addis Ababa [22], Rwanda [30], and Vietnam [19]. Experienced farmers may have gained practical knowledge on effective disease management, consequences of improper AMU, and the benefits of adhering to recommended protocols over time [41].

In this study, access to veterinary services was also found to be significantly associated with practices regarding AMU and AMR. This finding was consistent with previous studies conducted in Bangladesh [32], KwaZulu‐Natal, South Africa [40], and Zambia [38]. Access to veterinarians is beneficial for farmers because these professionals provide accurate diagnoses, proper drug prescriptions, and emphasize adherence to withdrawal periods, all of which promote prudent practices [32]. This underscores the critical importance of veterinary professional guidance in promoting responsible AMU.

One of the key strengths of this study is that it was conducted among poultry farm workers, offering valuable insights into their KAPs regarding AMU and resistance. The use of simple random sampling among poultry farm workers enhances the representativeness of the sample and supports the generalizability of the findings to the target population within the study areas. Furthermore, the use of digital data collection platforms and trained data collectors ensured high‐quality data that more accurately reflects real‐world conditions. However, this study has several limitations. First, the cross‐sectional nature of the study design precludes the establishment of definitive cause‐and‐effect relationships. Second, the use of self‐reported data to assess KAPs may have introduced social desirability bias, potentially affecting the accuracy of participants' responses. In addition, some subgroup categories, particularly certain educational‐status groups, had relatively small sample sizes, which may have reduced the precision of the effect estimates and resulted in wider confidence intervals. Therefore, the observed associations should be interpreted with appropriate caution.

## 5 Conclusion

This study revealed significant gaps in participants' KAPs regarding AMU and resistance in the study areas. Education level and farming experience were significantly associated with KAPs. In addition, access to poultry health information was significantly associated only with knowledge, whereas access to veterinary services was linked exclusively to practices. Therefore, it is crucial to enhance poultry farm workers' KAP through implementing regular training programs that focus on the responsible use of antimicrobials and also through improving access to poultry health information and services to minimize AMR challenges.

## Author Contributions

Mohamed Abdi was involved in conceptualization, data curation, formal analysis, investigation, methodology, software, validation, visualization, and writing – original draft. Jemal Mohammed was involved in supervision and writing – review and editing. Ukash Umer involved in supervision, writing – original draft, writing – review and editing. Gada Diba was involved in supervision, writing – review and editing. Fitsum Weldegebreal was involved in supervision, writing – review and editing. All authors have read and approved the final version of the manuscript. Ukash Umer had full access to all of the data in this study and takes complete responsibility for the integrity of the data and the accuracy of the data analysis.

## Conflicts of Interest

The authors declare that they have no conflicts of interest.

## Transparency Statement

The Ukash Umer affirms that this manuscript is an honest, accurate, and transparent account of the study being reported; that no important aspects of the study have been omitted; and that any discrepancies from the study as planned (and, if relevant, registered) have been explained.

## Data Availability

The authors confirm that all data underlying the results presented are available in the article.
